# Visual ordinal grading of aortic valve calcification on routine non-gated chest CT predicts prognosis and alters management

**DOI:** 10.1007/s00330-025-11553-w

**Published:** 2025-04-02

**Authors:** Samuel G. S. Gunning, John Graby, Yashesh Mody, Pia F. P. Charters, Tim A. Burnett, David Murphy, Ali Khavandi, Jonathan C. L. Rodrigues

**Affiliations:** 1 Department of Anaesthetics, Royal United Hospitals Bath NHS Foundation Trust, Avon, UK; 2 Department of Cardiology, Royal United Hospitals Bath NHS Foundation Trust, Avon, UK; 3 Department for Health, University of Bath, Bath, UK; 4 Department of Radiology, Royal United Hospitals Bath NHS Foundation Trust, Avon, UK

**Keywords:** Aortic valve stenosis, Aortic valve, calcification of, Tomography, X-ray computed, Echocardiography

## Abstract

**Objective:**

BSCI/BSTI guidelines recommend reporting aortic valve calcification (AVC) on all chest CTs regardless of indication. We assessed AVC frequency, severity, and association with aortic stenosis (AS) on echocardiography and its prognostic implications.

**Methods:**

Retrospective, single-centre analysis of consecutive chest CTs (January–December 2015) for 200 patients per age group (< 40, 40–49, 50–59, 60–69, 70–79, 80–89, ≥ 90) performed for medical, surgical, and oncological indications. CTs were re-reviewed for the presence and graded severity of AVC and coronary artery calcification (CAC). Corresponding echocardiography reports (within 5 years) reviewed for AS. Comorbidities and clinical outcomes were recorded.

**Results:**

One thousand three hundred seventy-seven patients were included (mean age 64 ± 20 years, 55% female). AVC was present in 25% (350/1377) and was more prevalent in males (*p* < 0.001). Frequency and severity increased with age (*p* < 0.001). 38% (524/1377) had an echocardiogram (median inter-test interval 4.3 months [IQR 0.4–17.5]). Sixteen per cent (29/178) with AVC had AS of any severity (8% [15/178] mild; 8% [14/178] moderate; 0% [0/178] severe). Sensitivity and specificity for AVC predicting AS were 91% and 70%, respectively. Extrapolating findings, 8% of individuals with AVC and without an echocardiogram may have undiagnosed AS. All-cause mortality occurred in 53% (734/1377), which AVC predicted independently of CAC and age (*p* < 0.001). Adjusting for confounders, severe AVC predicted all-cause mortality (HR 1.56 [1.10–2.22], *p* = 0.013).

**Conclusions:**

AVC identified AS in 16% of patients. Additionally, severe AVC is an independent predictor of all-cause mortality in multivariable analysis. Validation in a prospective cohort is required to inform clinical practice guidelines.

**Key Points:**

***Question*** *New guidelines recommend reporting AVC on all non-gated chest CTs, the prognostic and clinical relevance of which is uncertain*.

***Findings*** *There are associations between visually quantified AVC, AS on echocardiography, and all-cause mortality in an unselected population referred for routine chest CT*.

***Clinical relevance*** *These results support the reporting of all severities of AVC, especially severe AVC, as a prognostic marker in all age groups. The clinical implications require further clarification in a prospective cohort*.

**Graphical Abstract:**

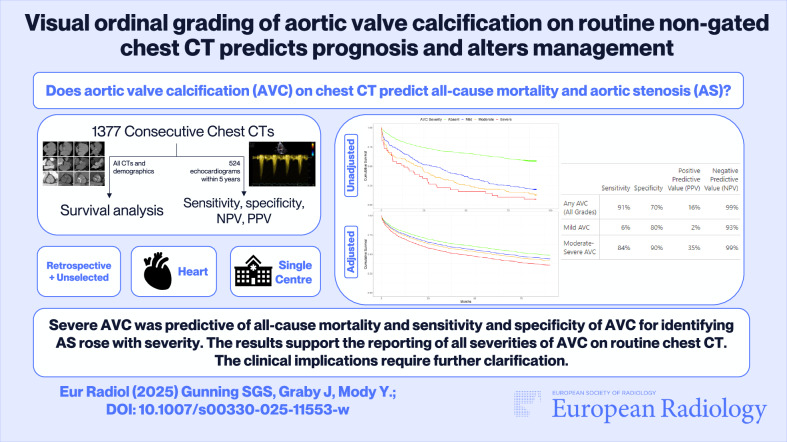

## Introduction

The global incidence of degenerative valvular heart disease is increasing, driven by growing and ageing populations [1]. It preferentially impacts developed nations with older patients [2]. Calcific aortic stenosis (AS) is the most prevalent valvular lesion, the progressive nature of which necessitates frequent surveillance. This is particularly true of moderate and severe grades, to ensure optimally timed intervention [3].

Prognosis declines as AS progresses such that, without aortic valve replacement (AVR), patients with severe AS have a 5-year mortality of over 50% [4]. Traditionally, AVR has been reserved for symptomatic, severe cases. However, emerging evidence on reduced procedural risks, heterogeneity of AS populations, and poor prognosis of moderate AS, supports consideration of earlier AVR in some asymptomatic and non-severe groups [5–7]. Testing this hypothesis in the severe, asymptomatic group is the subject of the ongoing EASY-AS randomized controlled trial [8]. Consequently, the population potentially eligible for intervention may expand, requiring novel approaches for the identification and surveillance of patients with AS.

Aortic valve calcification (AVC) is traditionally assessed via the Agatston method on unenhanced electrocardiogram (ECG)-gated CT, with AVC severity correlating closely with AS haemodynamic severity [9, 10]. Additionally, formal Agatston-style scoring of AVC on low-dose [9, 11] and non-ECG-gated CT [9, 11, 12] may correlate with scoring on ECG-gated CT, however, a visual ordinal assessment of AVC on non-ECG-gated CT is likely sufficient with close agreement between approaches [13].

AVC scoring via the Agatston method provides valuable prognostic information when assessing patients with AS. Severe AVC is strongly associated with severe AS and is linked to higher rates of AVR and mortality, independent of AS haemodynamics, patient demographics, and coronary artery calcification (CAC) [14, 15]. Additionally, quantifying AVC offers complementary diagnostic information in patients with incongruent echocardiography findings, informing the management of patients with low-flow, low-gradient AS [12]. Therefore, early identification of patients with AVC provides an opportunity to initiate investigation, surveillance, and treatment of those with AS. Consequently, a 2020 consensus statement from the British Society of Cardiovascular Imaging/British Society of Cardiac Computed Tomography (BSCI/BSCCT) and British Society of Thoracic Imaging (BSTI) recommends reporting and visually quantifying AVC (and CAC) on all non-contrast and contrast-enhanced, non-gated chest CT’s, irrespective of indication [16].

This study aimed to (1) assess the frequency and severity of AVC with visual quantification of calcific burden in an unselected patient cohort across all age groups undergoing routine non-gated chest CT imaging; (2) assess the relationship between AVC on CT and AS on echocardiography; and (3) evaluate the significance of AVC in predicting all-cause mortality.

## Methods

This retrospective observational study included patients who had previously undergone clinically indicated imaging and was approved as a service evaluation by our institution’s Trust Audit Committee. As per the Health Research Authority’s decision tool, written informed consent and ethical committee approval were not required [17].

### Study design

**Fig. 1:**
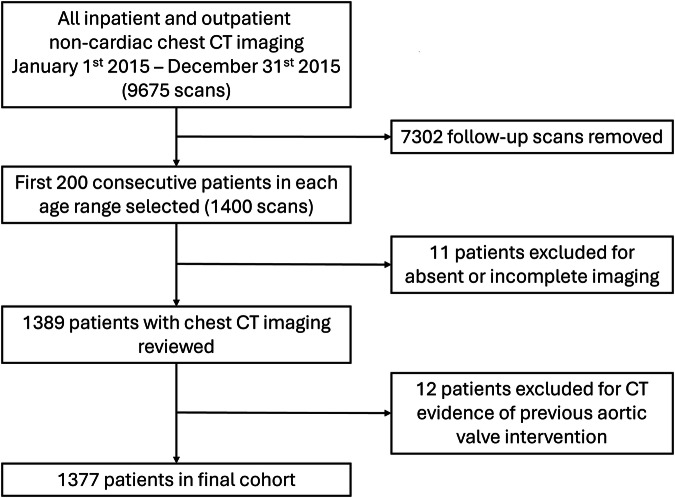
Study flowchart, with patients excluded if there was absent or incomplete chest imaging or if there was CT evidence of prior aortic valve intervention

From 1st January 2015, 200 consecutive non-gated chest CTs from each age group studied (< 40, 40–49, 50–59, 60–69, 70–79, 80–89 and ≥ 90) were included (excluding repeat and follow-up imaging [Fig. 1]). Scans were performed at The Royal United Hospital, Bath, in inpatient and outpatient settings on a 64-detector row CT machine (Siemens Edge, Siemens Healthineers) via a number of imaging protocols (Table S1) for a number of indications (Table S2). Following re-review, scans with evidence of previous valvular surgery or incomplete imaging data were excluded. To ensure conclusions best reflected real-world clinical practice, excluded scans were not replaced.

### AVC and CAC

All CTs were re-reviewed by one of two radiologists with 4 or more years of experience. They recorded the presence or absence of AVC and visually graded severity, assigning scores of 0 (none), 1 (mild), 2 (moderate), or 3 (severe), as per UK imaging recommendations [16]. All CTs were non-gated, preventing formal Agatston scoring. However, previous studies have demonstrated the reproducibility of this scoring technique, evidencing almost perfect agreement with Agatston scoring [13, 18].

As outlined by Graby et al, radiologists also reported the presence of CAC and visually quantified the degree of calcification using the same severity scale [19]. The four major epicardial coronary vessels were scored individually, and the summation of scores correlated with a total CAC severity (0 = none, 1–3 = mild, 4–8 = moderate, and 9–12 = severe).

### Electronic health records

Each patient’s electronic health record was reviewed for the presence of cardiovascular risk factors. Independently, all-cause mortality data and date of death were obtained via the National Health Service (NHS) Spine (the digital central information point for NHS systems).

### Echocardiography

The local reporting system was interrogated for an echocardiogram, for any indication, within 5 years on either side of each chest CT. If a patient had multiple echocardiograms within the defined time period, the scan assessing aortic valve morphology and function with the closest temporal relationship to the chest CT was selected. Focused echocardiograms without aortic valve assessment (e.g. to exclude chemotherapy-related cardiotoxicity) were excluded from the final analysis.

Data collected included the presence of aortic sclerosis and peak aortic valve gradient (mmHg). AS was graded in accordance with a combination of international guidelines by peak gradient into none (< 25 mmHg), mild (25–35 mmHg), moderate (36–63 mmHg), or severe (≥ 64 mmHg) [20, 21]. In cases where AVC was severe on CT and no AS was present on echocardiography, reports were closely interrogated for evidence of low-flow, low-gradient AS.

### Statistical analysis

Statistical analysis was performed with RStudio (v. 2022.12.0 Build 353; R v. 4.2.2). Continuous and categorical variables are expressed as mean (± standard deviation) or median (interquartile range [IQR]), and number (percentage), respectively. Differences were analysed using Chi-Squared or Fisher’s tests and the Wilcoxon rank-sum test. Multivariate logistic regression analysis facilitated age-adjusted analysis. Statistical significance was defined as *p* < 0.05.

Kaplan-Meier curves show time-to-event analysis. Follow-up was defined as the time from CT to the event (all-cause mortality) or censored on the 23rd of August 2022. Cox proportional hazards regression analyses were used to test and adjust for confounders. Due to limited events, and a wish to limit the risk of multicollinearity, only individual variables that significantly predicted both the exposure of interest (AVC on CT) and the outcome of interest (all-cause mortality) on univariate analysis were included as confounders in multivariate analysis.

Moderate and severe AVC were combined into a single category when calculating sensitivity, specificity, negative predictive value (NPV) and positive predictive value (PPV) of AVC on chest CT for AS on echocardiogram. This aligns with BSCI/BSCCT and BSTI guidelines, which recommend echocardiography in these categories alone.

Inter- and intra-observer variability in the reporting of AVC presence and severity was tested on 40 scans, re-reviewed by the same two radiologists, and blinded to baseline grading. Cohen’s κ was used for statistical testing. Pre-defined levels of agreement were used: ≤ 0 no agreement, 0.01–0.20 none to slight, 0.21–0.40 fair, 0.41– 0.60 moderate, 0.61–0.80 substantial, and 0.81–1.00 almost perfect agreement [22].

## Results

### Study population

One thousand three hundred seventy-seven patients with corresponding chest CT imaging (mean age 64 ± 20, 45% (614/1377) male; 68% (940/1377) contrast-enhanced CTs) were included in the analysis. A total of 23 patients were excluded, including 11 for incomplete imaging and 12 for CT evidence of prior valvular surgery. Demographics are summarised in Table 1.

**Table 1:** Study population demographics subdivided by AVC grade

		AVC grade				
Characteristic	Overall, *N* = 1377^a^	None, *N* = 1027^a^	Mild, *N* = 210^a^	Moderate, *N* = 99^a^	Severe, *N* = 41^a^	*p*-value^b^
Age						
Mean ± SD	64 ± 20	57 ± 18	80 ± 12	85 ± 8	86 ± 8	< 0.001
Median [IQR]	65 [48, 81]	56 [44, 71]	83 [73, 91]	87 [81, 90]	90 [82, 91]	< 0.001
Gender (male)	45% [614]	42% [429]	49% [102]	59% [58]	61% [25]	< 0.001
Diabetes mellitus	13% [185]	12% [125]	15% [31]	19% [19]	24% [10]	0.032
Cerebrovascular accident	4% [55]	3% [32]	6.2% [13]	6.1% [6]	9.8% [4]	0.017
Rheumatoid arthritis	2% [24]	2% [17]	1.4% [3]	1.0% [1]	7.3% [3]	0.11
Ischaemic heart disease	16% [221]	12% [123]	22% [47]	32% [32]	46% [19]	< 0.001
Atrial fibrillation/flutter	15% [205]	9% [91]	28% [59]	42% [42]	32% [13]	< 0.001
Hypertension	43% [596]	36% [370]	58% [121]	77% [76]	71% [29]	< 0.001
Chronic kidney disease	14% [187]	9% [90]	21% [44]	38% [38]	37% [15]	< 0.001
Dyslipidaemia	10% [143]	9% [89]	15% [31]	13% [13]	24% [10]	0.001
Obesity	20% [273]	19% [199]	18% [37]	32% [32]	12% [5]	0.007
Smoking history						
Never smoked	75% [1,031]	76% [779]	77% [161]	66% [65]	63% [26]	0.027
Ex-smoker	18% [246]	16% [168]	16% [34]	30% [30]	34% [14]	
Current smoker	7% [100]	8% [80]	7.1% [15]	4.0% [4]	2.4% [1]	
Family history of ischaemic heart disease	1% [11]	1% [10]	0.5% [1]	0% [0]	0% [0]	> 0.9
Cancer (as primary CT indication)	60% [829]	59% [608]	60% [127]	71% [70]	59% [24]	0.2

^a^ % [*n*]

^b^ Kruskal–Wallis rank sum test; Wilcoxon rank-sum test; Pearson’s Chi-squared test; and Fisher’s exact test

### Assessment of AVC

**Fig. 2:**
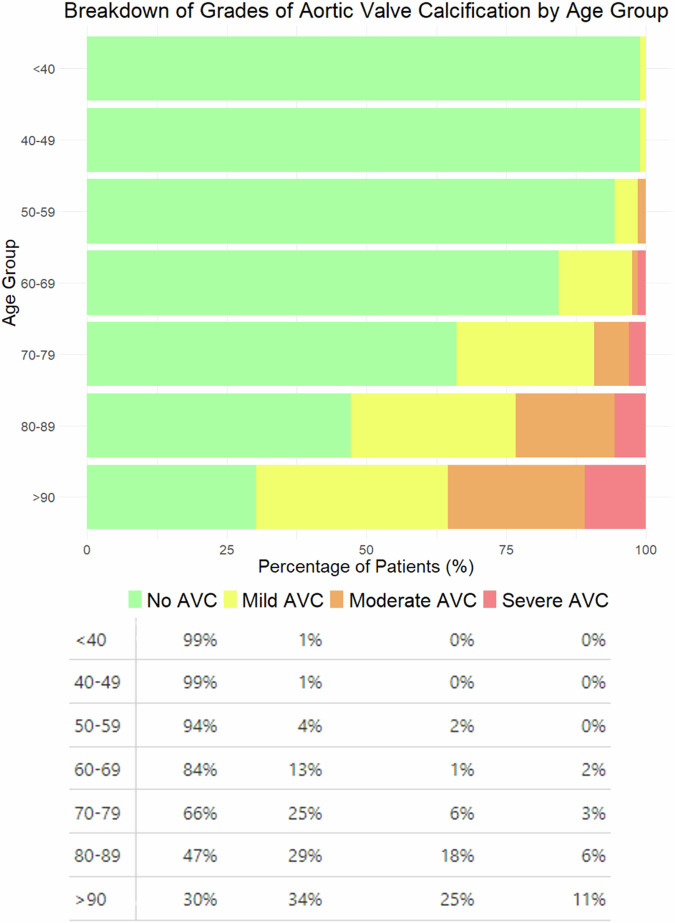
Frequency and severity (%) of AVC broken down by age group with percentages given

AVC was present in 25% (350/1377: 185/350 male, 165/350 female) of patients with a higher frequency in males (*p* < 0.001). Frequency increased with age, rising from 1% in the < 40 age group to 70% in the > 90 age group (*p* < 0.001; Fig. 2). Severity also increased with age with 90% (89/99) of moderate AVC and 88% (36/41) of severe AVC cases occurring in those aged over 75 (*p* < 0.001). AVC frequency and severity by age group are shown in Fig. 2.

CAC was present in 55% (764/1377) of patients. Frequency and severity of CAC also increased with age (*p* < 0.001) and were associated with AVC (*p* < 0.001), including when controlling for age (*p* = 0.004).

Intra-observer agreement was almost perfect for the presence (κ = 0.9, *p* < 0.001) and severity (κ = 0.9, *p* < 0.001) of AVC. Contrastingly, inter-observer agreement was moderate for the presence (κ = 0.5, *p* < 0.001) and severity (κ = 0.6, *p* < 0.001) of AVC.

### Corresponding echocardiography

Thirty-eight per cent (524/1377) of patients had an echocardiogram within 5 years of their CT scan (median time between tests 4.3 months [IQR 0.4–17.5; range 0–58.5]; 67% (351/524) within 1 year). A further 58 patients had focused echocardiograms with unknown AS status and were consequently excluded.

Sixteen per cent (29/178) of patients with AVC on chest CT, and a corresponding echocardiogram with aortic valve assessment, had AS of any severity (mild AS 8% [15/178]; moderate AS 8% [14/178]; severe AS 0% [0/178]). We identified incrementally more cases of AVC in patients with mild and moderate AS compared to no AS (mild AS *p* < 0.001; moderate AS *p* < 0.001). The severity of AS by the severity of AVC is shown in Table 2. There was no significant difference in median time to echocardiogram between groups with and without AVC (with AVC 5.6 months; without AVC 3.9 months, *p* = 0.163).

**Table 2:** Grade of AS on echocardiogram subdivided by grade of AVC on chest CT

	AS (echocardiography)				
Characteristic	None, *N* = 375^a^	Sclerosis, *N* = 117^a^	Mild, *N* = 16^a^	Moderate, *N* = 16^a^	Severe, *N* = 0^a^
AVC (CT)					
None	83% [310]	28% [33]	6% [1]	13% [2]	NA% [0]
Mild	14% [53]	39% [46]	13% [2]	0% [0]	NA% [0]
Moderate	3% [10]	27% [32]	25% [4]	25% [4]	NA% [0]
Severe	1% [2]	5% [6]	56% [9]	63% [10]	NA% [0]

^a^ % [*n*]

Forty-nine per cent (172/350) of patients had AVC on CT but no echocardiogram with aortic valve assessment within 5 years. Based on the frequency of AS on echocardiogram in patients with AVC on chest CT, this may equate to 8% (28/350) of patients with potentially undiagnosed AS (any severity).

Echocardiography identified AS in 1% (3/346) of patients without AVC on CT (mild AS 33% [1/3]; moderate AS 67% [2/3]; severe AS 0% [0/3]). Sixty-seven per cent (2/3) of these patients were female. Thirty-three per cent (1/3) had an aortic valve with bicuspid morphology. CT images for two patients with moderate AS and no AVC were re-interrogated, and in both, an element of non-calcific, fibrotic AS was suspected. CT identified 30% (8/27) of patients with severe AVC and no evidence of AS on echocardiogram. Interrogation of echocardiography reports revealed no cases of low-flow, low-gradient AS.

The sensitivity, specificity, PPV, and NPV of presence and severity of AVC on CT for AS on an echocardiogram within 5 years are shown in Table 3. A sub-analysis of echocardiograms within 1 year of chest CT demonstrated little disparity in these figures, supporting our extended interval analysis (Table S3).

**Table 3:** Specificity, sensitivity, PPV, and NPV, with confidence intervals, of presence and severities of AVC on chest CT for AS on echocardiogram within 5 years

	Sensitivity	Specificity	PPV	NPV
Any AVC (all grades)	91% [81, 100]	70% [66, 74]	16% [11, 22]	99% [98, 100]
	TP: 29 FP: 149 TN: 343 FN: 3			
Mild AVC	6% [0, 15]	80% [76, 84]	2% [0, 5]	93% [90, 95]
	TP: 2 FP: 99 TN: 393 FN: 30			
Moderate-severe AVC	84% [72, 97]	90% [87, 93]	35% [24, 46]	99% [98, 100]
	TP: 27 FP: 50 TN: 442 FN: 5			

True positives (TP), false positives (FP), true negatives (TN), and false negatives (FN) are also given for each grade of AVC

*%* [95% CI], *TP* true positive, *FP* false positive, *TN* true negative, *FN* false negative

Further sub-analysis by age, dividing our cohort into two groups (over and under 75-years-old), is shown in Table 4.

**Table 4:** Sensitivity and specificity, with confidence intervals, for presence and severities of AVC on chest CT for AS on echocardiogram for patients aged under 75 and 75 or over

	Under 75		Over 75	
	Sensitivity	Specificity	Sensitivity	Specificity
Any AVC (all grades)	80% [45, 100]	88% [84, 91]	93% [83, 100]	42% [35, 49]
Mild AVC	0% [0, 0]	89% [86, 93]	7% [0, 17]	65% [59, 72]
Moderate-severe AVC	80% [45, 100]	98% [97, 100]	85% [72, 99]	77% [71, 83]

% [95% CI]

### Clinical outcomes

Fifty-three per cent (734/1377) of patients died during follow-up (median follow-up 74 months, [IQR 14.6–90.5]), rising with age (Table S4). The presence of AVC on CT was associated with all-cause mortality, as was the severity of AVC (any AVC *p* < 0.001; mild AVC *p* < 0.001; moderate AVC *p* < 0.001; severe AVC *p* < 0.001). So too were the presence and severity of CAC (any CAC *p* < 0.001; mild CAC *p* < 0.001; moderate CAC *p* < 0.001; severe CAC *p* < 0.001) and both presence and severity of AS on echocardiogram (any AS *p* < 0.001; mild AS *p* < 0.001; moderate AS *p* = 0.005). Table S5 in the supplementary data shows the distribution of deceased patients by these predictors. All severities of AVC independently predicted all-cause mortality following adjustment for severity of CAC (mild AVC HR 2.07 [1.71–2.49], *p* < 0.001; moderate AVC HR 2.64 [2.07–3.36], *p* < 0.001; severe AVC HR 3.29 [2.34–4.61], *p* < 0.001) and age (mild AVC HR 1.22 [1.00–1.48], *p* = 0.048; moderate AVC HR 1.36 [1.06–1.74], *p* = 0.016; severe AVC HR 1.67 [1.18–2.35], *p* = 0.004).

**Fig. 3:**
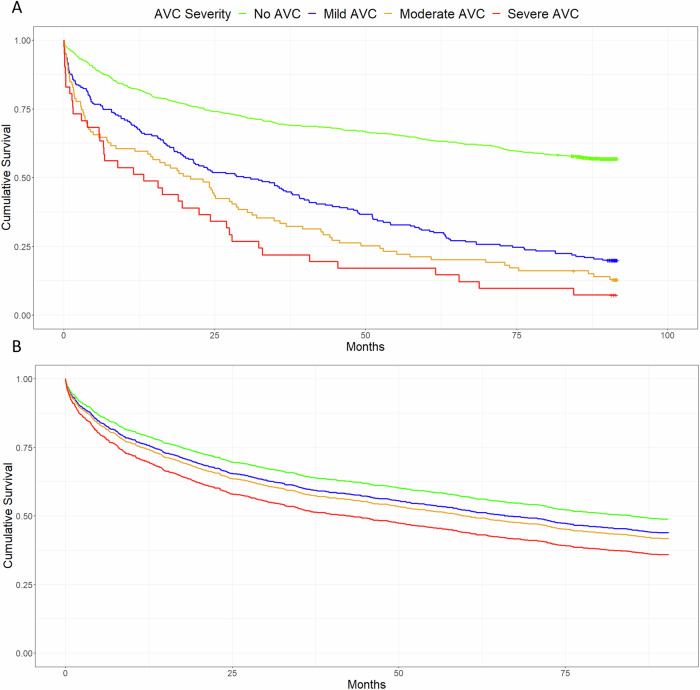
**A** Unadjusted survival curves for AVC severity and all-cause mortality. **B** Adjusted survival curves for AVC severity and all-cause mortality

**Fig. 4:**
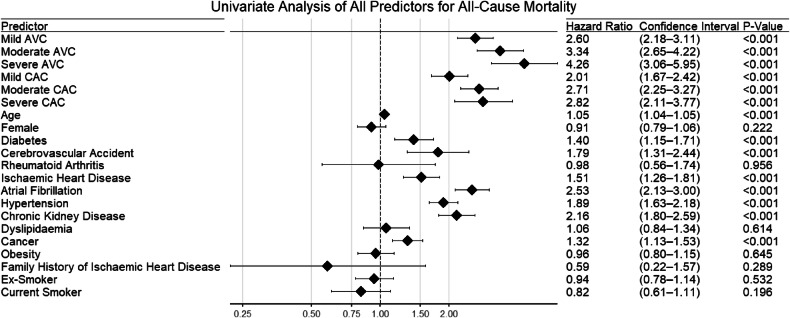
Univariate analysis of all predictor variables for all-cause mortality

**Fig. 5:**
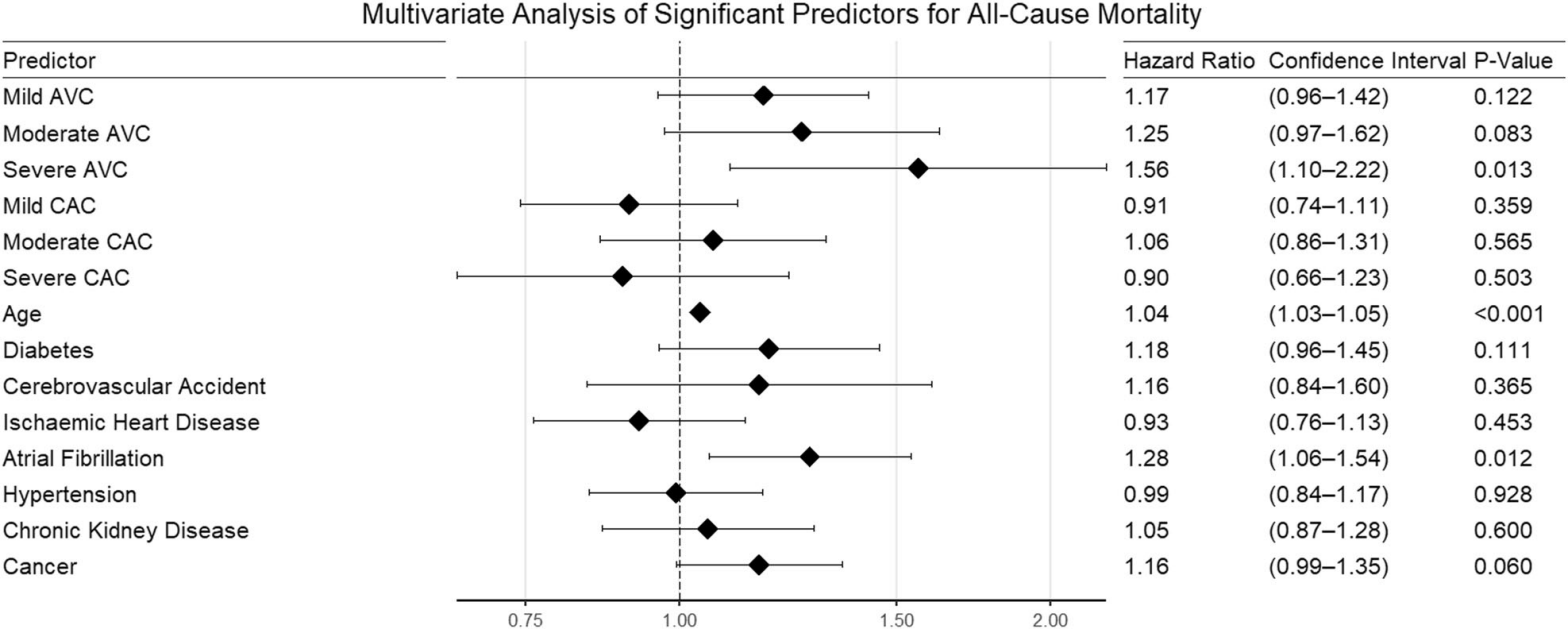
Multivariate analysis for all significant predictor variables for all-cause mortality

After adjusting for multiple confounders, only severe AVC predicted all-cause mortality (confounders: severity of CAC on CT, age, diabetes mellitus, atrial fibrillation, previous cerebrovascular accident, pre-existing diagnosis of ischaemic heart disease, hypertension, chronic kidney disease, and cancer as the primary scan indication; severe AVC HR 1.56 [1.10–2.22], *p* = 0.013). Original and adjusted survival curves are provided in Fig. 3. Forest plots for univariate and multivariate analyses are provided in Figs. 4 and 5.

## Discussion

This study provides an up-to-date assessment of the frequency and severity of AVC when visually quantifying calcific burden in unselected patients across all age groups undergoing routine, non-gated CT chest imaging [23]. Applying ordinal grading of AVC, as recommended by BSCI/BSCCT and BSTI, we demonstrate the potential prognostic and management implications for the reporting of all grades of AVC on CT chests, across age groups.

In our study, AVC was present in 25% of 1377 patients. These results differ from previous studies assessing AVC both formally with Agatston scoring and visually, where the frequency was 13% of 1812 participants [24] and 12% of 1225 participants [13], respectively. The witnessed disparity in frequency likely reflects differences in baseline cohort characteristics, especially age, which was greater in our study (64 ± 20 years [range 18–102]) compared to the two referenced studies (59 ± 16 [range 20–86] and 60 ± 9 [range not given] years, respectively). The selection of 200 participants from each age group has created a uniformly distributed population with a large age range, and it is therefore inferred that our population has a greater proportion of older patients than previous studies, with age significantly associated with both frequency and severity of AVC. We hypothesise, that the high overall mortality rate observed in our population is likely a result of this selection strategy in combination with a high number of patients undergoing CT imaging to exclude, stage, or surveil malignancies.

The association between AVC and CAC, suggests a common underlying pathophysiological process with calcification of the aortic valve and coronary arteries likely reflecting more generalised calcification of systemic vasculature [15]. Additionally, following adjustment for confounding factors including CAC, severe AVC was independently associated with all-cause mortality. This aligns with previous research suggesting AVC has independent predictive value for a patient’s general atherosclerotic risk [9]. The independent predictive power of both AVC and CAC, as well as other previously evidenced extra-coronary calcification, may suggest it is the underlying pathophysiological mechanism, diffuse atherosclerosis propagating systemic, pro-inflammatory pathways rather than localised calcification, which predicts mortality [19, 25].

The presence of AVC was associated with the frequency and severity of AS on echocardiography. Conversely, the presence of AVC on CT does not equate to the presence of AS on echocardiogram, even in severe cases. This is likely a product of CT false positives, difficulty differentiating aortic valve annular and cusp calcification on non-gated chest CT, and imperfect sensitivity and specificity intrinsic to echocardiography [26]. As such, whilst reported AVC acts as a potential biomarker for the presence of AS, it is not pathognomonic and requires clinical corroboration for an informed referral for echocardiography. If referred for echocardiography based on chest CT findings alone, it would be prudent to inform those patients that only approximately 1 in 6 of those with AVC will have AS and require surveillance.

Our study identified 2 cases of AS in 101 (2%) patients with mild AVC on CT and a corresponding echocardiogram. According to BSCI/BSCCT and BSTI guidelines, which only recommend echocardiography in patients with moderate or severe AVC, these cases would be missed. Furthermore, extrapolating our figures on the frequency of AS in the subgroup with AVC, we may expect to have detected an additional 29 cases of AS (any severity) in those without an echocardiogram. This equates to just over 1 in 12 of those with AVC in our sample and outlines the role AVC reporting may play in the earlier identification of asymptomatic AS, which is also associated with increased cardiovascular risk. This is especially relevant given the move towards targeted earlier intervention for AS in appropriate cases [5–7].

Whilst the prognostic benefit of incidental AVC identification is evident, the clinical value realised through surveillance of patients with previously unidentified AS is more ambiguous. Reporting incidental AVC would increase the detection of asymptomatic AS, but as demonstrated in our population, these cases will most likely be mild, and very rarely severe, currently the only group requiring intervention. Recent results of the early valve replacement guided by biomarkers of LV decompensation in asymptomatic patients with severe AS trial suggest that even patients with asymptomatic severe AS, who show evidence of myocardial damage potentially indicative of more advanced disease, are unlikely to benefit from early intervention [27]. These findings further question the benefit of surveillance in this patient group, although the potential advantages of early intervention remain uncertain for patients with asymptomatic severe AS who do not have evidence of cardiac fibrosis. Consequently, we do not necessarily suggest an early intervention in these patients but simply a mechanism to augment case findings of AS that can be followed up and intervened upon when the risk-benefit ratio is appropriate.

Compounding this is the relatively low sensitivity of AVC on CT for AS on echocardiography. Non-calcific, fibrotic AS, more common in younger female patients or those with bicuspid aortic valve, and potentially present in 3 of 346 patients without AVC and with an echocardiogram, by its very nature, limits the sensitivity of AVC [28]. However, increasing specificity with worsening AVC severity supports the BSCI/BSCCT and BSTI recommendation for echocardiography in moderate and severe AVC populations and, if we acknowledge the value of identifying mild AS for enabling surveillance of patients who may need future intervention, then extending reporting to include mild AVC may benefit up to 6% of patients. Further research into the advantages and the potential burden on both patients and health systems, especially echocardiography services, of broadening AS surveillance is required in order to fully understand the impact of reporting incidental AVC.

Age-stratified sub-analysis demonstrates the importance of contextualising chest CT findings. Whereas the absence of AVC is reassuring in younger patients, the relatively low sensitivity, potentially reflecting higher rates of non-calcific AS, limits the value of CT as a screening test for AS in this age group. Comparatively, CT demonstrates its predictive value for AS in older patients.

Irrespective of age group, contextualisation of CT findings, based on scan indication and patient factors, is essential in ensuring appropriate further investigation. Notably, the incorporation of a frailty score alongside or as an alternative to age, thereby substituting chronological age for biological age, may improve our assessment of those requiring echocardiography. However, this will first require validation in the literature. For the time being, the poor prognosis of severe AS means that if AVC on chest CT can facilitate AS diagnosis, its routine reporting can aid personalised patient risk stratification.

This study is limited by its retrospective, single-centre nature. This is unlikely to account for geographical variations in population characteristics and associated pathological patterns. Additionally, our assessment of the likelihood of AVC detecting AS is caveated by the lack of defined contemporaneous CT imaging and echocardiography. Having selected only the closest complete echocardiogram, we cannot comment on the likelihood and timing of developing AS given the grade of AVC identified on CT. However, our data provides a pragmatic estimation of the real-world likelihood of detecting AS over a 5-year period in the context of CT findings. This timeframe was deemed reasonable given the relatively slow, progressive nature of AS.

We lack data on pre-existing AS diagnosis and echocardiography indication, and are subsequently unable to conclude how many new cases of AS would have been identified following CT. Extrapolated figures for undiagnosed AS in the population with AVC and no echocardiogram are likely overestimations if echocardiography has been requested anticipating AS for reasons separate from incidental AVC on chest CT. Clinical recommendations are therefore avoided. However, routinely reporting AVC on chest CT would allow clinicians to assess for a history and signs of AS, informing the decision as to the need for an echocardiogram. This practice would be further supported by additional research in a large cohort validating the reproducibility of visual ordinal calcification grading which, although reduced in our study (potentially as a result of misidentification of aortic annulus calcification) has previously been shown to have substantial reproducibility [13].

Our study demonstrates the value of reporting AVC on routine chest CT based on the identification of patients with some degree of AS and highlights the additional cardiovascular risk carried by patients with AVC. Further prospective, multi-centre work, complemented by supplementary symptom, comorbidity, frailty and imaging data, is required to validate our conclusions and test the true clinical impact of reporting AVC as per BSCI/BSCCT and BSTI guidelines. This should include an evaluation of the potential effect that adherence to these recommendations may have on clinical management and NHS resource use. This research will prove essential in validating these recommendations and contributing to the evidence base required for widespread guideline adherence.

## Conclusion

AVC on routine non-cardiac chest CT is prevalent in 25% of our stratified study population and rises with age. Both the presence of AVC and visual ordinal assessment of severity were associated with the presence of AS and predicted all-cause mortality independent of age and CAC. Severe AVC reported on non-gated CT thorax is an independent predictor of all-cause mortality, and validation in a prospective observational study is required to inform clinical practice guidelines.

## Supplementary information

Supplementary information

## Data Availability

The data underlying this article cannot be shared publicly for the privacy of individuals who participated in the study. The data may be shared on reasonable request to the corresponding author.

## Abbreviations

AS: Aortic stenosis
AVC: Aortic valve calcification
AVR: Aortic valve replacement
BSCCT: British Society of Cardiac Computed Tomography
BSCI: British Society of Cardiovascular Imaging
BSTI: British Society of Thoracic Imaging
CAC: Coronary artery calcification
CT: Computed tomography
EASY-AS: Early valve replacement in severe asymptomatic aortic stenosis
ECG: Electrocardiogram
IQR: Interquartile range
NHS: National Health Service
NPV: Negative predictive value
PPV: Positive predictive value
