# Supplementary material for: Visual ordinal grading of aortic valve calcification on routine non-gated chest CT predicts prognosis and alters management
Source: Eur Radiol. 2025 Apr 2;35(10):6291–301. doi: 10.1007/s00330-025-11553-w (PMC12417276; doi:10.1007/s00330-025-11553-w)
Supplement: Supplementary file 1 — Supplementary information [file 330_2025_11553_MOESM1_ESM.pdf]

Visual ordinal grading of aortic valve calcification on routine non-gated chest CT predicts prognosis

ELECTRONIC SUPPLEMENTARY MATERIAL

Eur Radiol (2025) Gunning SGS, Graby J, Mody Y, et al.

Table S1. CT scan acquisition type sub-divided by age group and presence or absence of aortic valve calcification (AVC) on CT. (CTPA = CT pulmonary angiogram; CT C/A/P (c) = CT chest/abdomen/pelvis with contrast; CT C/A/P (nc) = CT chest/abdomen/pelvis without contrast; CT C/A (c) = CT chest/abdomen with contrast; CT C/A (nc) = CT chest/abdomen without contrast; HRCT = high resolution CT).

		**<40 Years**		**40-49 Years**		**50-59 Years**		**60-69 Years**		**70-79 Years**		**80-89 Years**		**>90 Years**	
	**Overall**, N = 1377	**AVC-**, N = 197[1]	**AVC+**, N = 2[1]	**AVC-**, N = 198[1]	**AVC+**, N = 2[1]	**AVC-**, N = 185[1]	**AVC+**, N = 11[1]	**AVC-**, N = 167[1]	**AVC+**, N = 31[1]	**AVC-**, N = 129[1]	**AVC+**, N = 66[1]	**AVC-**, N = 93[1]	**AVC+**, N = 104[1]	**AVC-**, N = 58[1]	**AVC+**, N = 134[1]
**Examination**															
CTPA	21% [288]	25% [50]	0% [0]	20% [40]	0% [0]	17% [32]	55% [6]	18% [30]	9.7% [3]	20% [26]	12% [8]	23% [21]	18% [19]	29% [17]	27% [36]
CT C/A/P (c)	33% [452]	26% [51]	100% [2]	39% [77]	0% [0]	36% [66]	18% [2]	41% [68]	32% [10]	40% [51]	39% [26]	31% [29]	29% [30]	24% [14]	19% [26]
CT C/A/P (nc)	10% [144]	11% [21]	0% [0]	6.1% [12]	0% [0]	7.6% [14]	0% [0]	4.2% [7]	6.5% [2]	9.3% [12]	9.1% [6]	9.7% [9]	13% [13]	21% [12]	27% [36]
CT C/A (c)	4.1% [56]	1.5% [3]	0% [0]	3.0% [6]	0% [0]	5.4% [10]	9.1% [1]	7.2% [12]	0% [0]	2.3% [3]	7.6% [5]	4.3% [4]	4.8% [5]	6.9% [4]	2.2% [3]
CT C/A (nc)	0.9% [12]	1.0% [2]	0% [0]	0% [0]	0% [0]	1.6% [3]	0% [0]	0.6% [1]	0% [0]	0% [0]	1.5% [1]	1.1% [1]	1.0% [1]	1.7% [1]	1.5% [2]
CT Chest (c)	10% [144]	9.6% [19]	0% [0]	13% [25]	50% [1]	9.7% [18]	9.1% [1]	13% [22]	13% [4]	7.0% [9]	7.6% [5]	13% [12]	13% [14]	3.4% [2]	9.0% [12]
CT Chest (nc)	15% [203]	15% [30]	0% [0]	11% [21]	50% [1]	18% [33]	0% [0]	12% [20]	29% [9]	16% [20]	23% [15]	12% [11]	19% [20]	12% [7]	12% [16]
HRCT	5.7% [78]	11% [21]	0% [0]	8.6% [17]	0% [0]	4.9% [9]	9.1% [1]	4.2% [7]	9.7% [3]	6.2% [8]	0% [0]	6.5% [6]	1.9% [2]	1.7% [1]	2.2% [3]

[1]%[n]

Table S2. CT scan indication sub-divided by age group. Scans requested for diagnosis, staging or surveillance of cancer are separated, as are scans for medical and surgical indications.

	**Overall**, N = 1377[1]	**<40**, N = 199[1]	**40-49**, N = 200[1]	**50-59**, N = 196[1]	**60-69**, N = 198[1]	**70-79**, N = 195[1]	**80-89**, N = 197[1]	**>90**, N = 192[1]
**CT Indication**								
Cancer	60% [820]	46% [91]	59% [118]	69% [132]	65% [127]	63% [122]	60% [116]	62% [114]
Non-Cancer	33% [448]	51% [102]	36% [71]	28% [55]	29% [57]	26% [51]	32% [63]	26% [49]
Medical	29% [397]	41% [82]	32% [64]	26% [51]	27% [54]	25% [48]	29% [58]	21% [40]
PE	19% [257]	23% [46]	19% [38]	18% [35]	19% [36]	16% [32]	21% [40]	16% [30]
Chronic Lung Disease	5% [74]	9% [18]	8% [16]	6% [12]	4% [8]	4% [8]	4% [8]	2% [4]
Infection	4% [56]	7% [14]	4% [8]	2% [4]	5% [9]	4% [8]	4% [8]	3% [5]
Other Medical	1% [10]	2% [4]	1% [2]	0% [0]	1% [1]	0% [0]	1% [2]	1% [1]
Surgical	4% [51]	10% [20]	4% [7]	2% [4]	2% [3]	2% [3]	3% [5]	5% [9]
Abdominal Surgical Issue	1% [12]	1% [2]	2% [3]	1% [2]	0% [0]	1% [2]	1% [2]	1% [1]
Trauma	2% [34]	9% [17]	2% [3]	1% [2]	1% [1]	1% [1]	2% [3]	4% [7]
Other Surgical	0.4% [5]	1% [1]	1% [1]	0% [0]	1% [2]	0% [0]	0% [0]	1% [1]
Unknown	8% [109]	3% [6]	6% [11]	5% [9]	7% [14]	11% [22]	9% [18]	15% [29]

[1] % [n]

Table S3. Sub-analysis demonstrating sensitivity, specificity, PPV and NPV, with confidence intervals, for presence and severity of AVC on CT for AS on echocardiogram within 1 year. True positives (TP), false positive (FP), true negatives (TN) and false negatives (FN) also given for each grade of AVC.

	Sensitivity	Specificity	Positive Predictive Value (PPV)	Negative Predictive Value (NPV)
Any AVC (All Grades)	88% [76, 100]	72% [67, 77]	20% [13, 28]	99% [97, 100]
	TP: 23 FP: 90 TN: 235 FN: 3			
Mild AVC	8% [0, 18]	82% [77, 86]	3% [0, 8]	92% [89, 95]
	TP: 2 FP: 60 TN: 265 FN: 24			
Moderate-Severe AVC	81% [66, 96]	91% [88, 94]	41% [28, 55]	98% [97, 100]
	TP: 21 FP: 30 TN: 295 FN: 5			

% [95% CI]; TP = true positive, FP = false positive, TN = true negative, FN = false negative

Table S4. Mortality and median follow-up duration sub-divided by age group.

Age group (years)	Total number of patients (N)	Died (n)	Median follow-up duration (months [IQR])
**<40**	199	32	87.5 [85.4 – 89.5]
**40-49**	200	51	88.8 [82.2 – 90.4]
**50-59**	196	81	90.2 [29.2 – 91.1]
**60-69**	198	113	70.0 [15.0 – 91.1]
**70-79**	195	114	66.5 [15.9 – 84.5]
**80-89**	197	156	34.1 [9.0 – 84.5]
**>90**	192	187	10.4 [1.6 – 31.1]

Table S5. Distribution of patients, alive and dead, by age, AVC presence and severity, AS severity and CAC presence.

**Characteristic**	**Alive**, N = 643[1]	**Dead**, N = 734[1]	**Overall**, N = 1,377[1]
Age			
Mean ± SD	52 ± 17	74 ± 17	64 ± 20
Median [IQR]	50 [39, 66]	78 [62, 90]	65 [48, 81]
Aortic Valve Calcification	9% [58]	40% [292]	25% [350]
Grade of Aortic Valve Calcification			
None	91% [585]	60% [442]	75% [1,027]
Mild	7% [42]	23% [168]	15% [210]
Moderate	2% [13]	12% [86]	7% [99]
Severe	1% [3]	5% [38]	3% [41]
Grade of Aortic Stenosis			
None	99% [203]	91% [289]	94% [492]
Mild	0% [0]	5% [16]	3% [16]
Moderate	1% [2]	4% [14]	3% [16]
Severe	0% [0]	0% [0]	0% [0]
Unknown	438	415	853
Grade of Coronary Artery Calcification			
None	60% [390]	32% [233]	45% [623]
Mild	24% [150]	31% [227]	27% [377]
Moderate	13% [83]	30% [216]	22% [299]
Severe	3% [20]	8% [58]	6% [78]

[1] % [n]
